# Orientation Transfer in Vernier and Stereoacuity Training

**DOI:** 10.1371/journal.pone.0145770

**Published:** 2015-12-23

**Authors:** Nathaniel Snell, Florian Kattner, Bas Rokers, C. Shawn Green

**Affiliations:** 1 Department of Psychology, University of Wisconsin-Madison, Madison, WI, United States of America; 2 Department of Neuroscience, Brown University, Providence, Rhode Island, United States of America; Durham University, UNITED KINGDOM

## Abstract

Human performance on various visual tasks can be improved substantially via training. However, the enhancements are frequently specific to relatively low-level stimulus dimensions. While such specificity has often been thought to be indicative of a low-level neural locus of learning, recent research suggests that these same effects can be accounted for by changes in higher-level areas–in particular in the way higher-level areas read out information from lower-level areas in the service of highly practiced decisions. Here we contrast the degree of orientation transfer seen after training on two different tasks—vernier acuity and stereoacuity. Importantly, while the decision rule that could improve vernier acuity (i.e. a discriminant in the image plane) would not be transferable across orientations, the simplest rule that could be learned to solve the stereoacuity task (i.e. a discriminant in the depth plane) would be insensitive to changes in orientation. Thus, given a read-out hypothesis, more substantial transfer would be expected as a result of stereoacuity than vernier acuity training. To test this prediction, participants were trained (7500 total trials) on either a stereoacuity (*N* = 9) or vernier acuity (*N* = 7) task with the stimuli in either a vertical or horizontal configuration (balanced across participants). Following training, transfer to the untrained orientation was assessed. As predicted, evidence for relatively orientation specific learning was observed in vernier trained participants, while no evidence of specificity was observed in stereo trained participants. These results build upon the emerging view that perceptual learning (even very specific learning effects) may reflect changes in inferences made by high-level areas, rather than necessarily fully reflecting changes in the receptive field properties of low-level areas.

## Introduction

Humans are excellent learners in the visual domain. With proper training (e.g. suitable numbers of trials, spacing between learning sessions, feedback if necessary, initial starting conditions and ramping up of difficulty), performance on any number of tasks thought to tap basic visual abilities can be substantially improved. Such results have been seen on tasks measuring sensitivity to basic stimulus features such as orientation [1], contrast [2], texture [3], spatial frequency [1], motion direction [4] and motion speed [5]. Strong learning is seen even in tasks where untrained human vision is already exceptionally good–into the hyperacuity range (e.g. vernier acuity and stereoacuity [6]).

However, while it is true that training can produce impressive enhancements on the trained task itself, such improvements typically fail to transfer when even seemingly minor changes are made to the experimental setup [7]. For instance, Fiorentini and Berardi [1] trained participants to discriminate between two complex vertical gratings. Although participant performance rose from near chance levels to near ceiling levels over the course of training, no benefit of this training was seen either when the orientation of the stimuli was rotated by 90° or when the spatial frequency was changed (either halved or doubled). Similarly, Karni and Sagi [3] showed that improvements in identifying the orientation of a texture patch presented in one location in the visual field were abolished when participants were tested on the exact same stimuli in a different retinal location. To some extent, specificity of learning has been observed for nearly every dimension where learning has been observed: e.g. orientation, spatial frequency, contrast, retinal location, motion direction, motion speed, luminance, etc. [8].

In one highly influential framework in the field, such specificity of learning has been considered as a potential marker of the neural locus of the learning effects. Under what has been dubbed “psycho-anatomy” logic [9], learning that is specific to, for instance, stimulus orientation, would be best attributed to changes in early visual areas (i.e. V1) where receptive fields are strongly orientation selective. Similarly, learning that is specific for the trained eye would potentially be attributed to even earlier areas (e.g. LGN), where cells respond to information only from one eye or the other. Transfer of learning, in such a view, is dependent on factors such as the difficulty of the trained task (although see [10]). The relatively larger and less finely tuned receptive fields of higher visual areas are capable of handling easier tasks, which in turn engenders more transfer [11]. As tasks become more difficult, successful performance necessitates a move down the visual hierarchy to cells with higher signal-to-noise ratios. The increased selectivity of the cells in this area means that less transfer will be observed to untrained dimensions.

An alternative framework suggests that stimulus- and/or task-specific learning need not necessarily reflect changes in receptive field properties in early visual areas, but instead that these improvements could be at least partially mediated by enhancements in the ability of higher-level areas to read-out the information provided by lower-level areas in the service of a specific decision [12–15]. Thus, in this view, stimulus-specific learning could reflect the reweighting of connections between low-level visual areas and integration-level areas. Evidence supporting this framework has come from behavioral studies, brain imaging, and single cell recordings. For example, the degree and type of learning transfer seen after “double-training” experiments (i.e. subjects who are trained on a contrast discrimination task at location #1 and then a totally different orientation discrimination task at location #2 show full transfer of their initial contrast discrimination learning to location #2 - [2]) and “training plus exposure” experiments (wherein observers show transfer after having been passively exposed to relevant transfer dimensions–[13]) cannot be well explained via classic models that posit an entirely low-level locus for perceptual learning. Instead, the results are consistent with a model wherein higher-level decision units learn a set of rules for weighting inputs from low-level areas. These rules can then potentially be applied to untrained stimuli (e.g. new orientations or retinal locations), assuming the training experience has allowed for the necessary functional connections to be formed (see also [16, 17]). Consistent with these behavioral results, a neuroimaging study by Kahnt and colleagues [18] found that while information about stimulus orientation was present in both early visual and higher cortical regions, it was changes in the higher-level areas outside visual cortex that best tracked the behavioral improvements that occurred via training. A similar pattern of data has also been observed in single-cell recording studies in non-human primates where behavioral improvements in motion direction discrimination were associated with changes in integration/decision-making brain areas (lateral intraparietal area–LIP) rather than in more purely sensory areas (i.e. MT—[12, 19, 20]).

**Fig 1:**
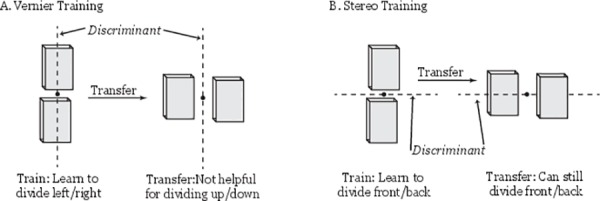
Orientation specificity predicted for vernier, but not stereoacuity training. A) The simplest rule that can be learned to perform the vernier acuity task is a simple discriminant function. A participant trained with the stimuli in a vertical orientation would thus learn a discriminant to divide the space into “left” and “right” answers. This rule is of little use when the stimuli are rotated by 90° as the critical comparison is now related to “above” or “below”. B) Conversely, essentially the same rule can be used regardless of stimulus orientation in the stereoacuity task (i.e. the discriminant that divides “front” from “back” does not depend on whether the stimuli are oriented vertically or horizontally.*Note–the stimuli in the current experiment were flat squares– 3D boxes are presented above for demonstration purposes.

Here we contrast the degree of orientation specificity that arises from training on two hyperacuity tasks–a vernier acuity task and a stereoacuity task. Given the framework above, improvements in both tasks could be explained by participants refining a reasonably simple rule, corresponding to a decision boundary or discriminant in the stimulus feature space. We hypothesized that although the minimal rule that could be learned to solve a vernier task would be of little use when transferring across orientations (as the discriminant that best separates vertical stimuli is of no help in separating horizontal stimuli), the minimal rule that could be learned to solve a stereoacuity task would be of considerable use when transferring across orientations (as a very similar, if not identical discriminant could be utilized–see Fig 1). This would then predict that orientation transfer would be observed in stereo-trained subjects, but not in vernier-trained subjects (though some degree of specificity may be observed even in the stereoacuity condition–e.g. see [6]).

To test our hypothesis, we first measured participants’ vernier and stereoacuity thresholds using stimuli in both horizontal and vertical orientations. Individual participants were then trained on one combination of task and orientation for five sessions. In order to ensure that any differences in transfer could not be attributed to differences in visual experience, all subjects viewed the same stimuli across training- that is, all stimuli were offset in both the image-plane and in the depth-plane. We asked the participants’ to report decisions depending on their assigned task: vernier = image-plane, stereo = depth-plane. Finally, a post-test identical to the pre-test was administered to assess transfer both across orientation within the trained task, as well as across tasks. As has been repeatedly observed [7], no significant orientation transfer was observed in the participants trained on the vernier acuity task. However, as predicted, significant orientation transfer was observed in the stereo acuity trained group.

## Methods

### Ethics statement

This research was approved by the University of Wisconsin-Madison Education and Social/Behavioral Sciences Institutional Review Board. All participants provided written informed consent prior to participation.

### Participants

Twenty-eight participants, all with normal or corrected-to-normal visual acuity, were initially enrolled in the study. Because the hypotheses in question rely on the comparison of pre-test and post-test thresholds, only participants with a pre-test stereothreshold within the range tested (i.e. thresholds < 120 arcsec) were allowed to continue into the training phase. Twelve participants failed to reach this level of pre-test performance and thus did not continue in the experiment (all twelve failed to reach threshold performance on either one or both of the stereoacuity pre-tests). Of the sixteen participants that continued into the training phase of the study, seven were assigned to the vernier training group (see description below), while nine were assigned to the stereo training group. Fifteen of the participants were naïve to psychophysical studies; one participant was an experienced psychophysical viewer. All participants were uninformed as to the purpose of the study.

### Apparatus and display

The stimuli were generated on an Apple Mac Pro computer running Windows 7 using Matlab (Mathworks, Natick, MA, USA) and the Psychophysics Toolbox 3 [21, 22, 23].

Stimuli were presented on a 23”-wide Planar SA2311W monitor with a resolution of 1920x1080 pixels and a refresh rate of 120 Hz. Participants viewed the screen while wearing NVIDIA 3D Vision shutter glasses. These glasses use temporal interleaving of frames to create a three-dimensional percept, and therefore the effective refresh rate was 60 Hz per eye. Participants viewed the stimuli from a distance of 23.84 m with their heads held in fixed position with a chinrest. The relatively long viewing distance was achieved through a mirror setup wherein two front-surface mirrors were placed on opposite ends of the room and the observer viewed the display through its reflected image (note: the participants’ direct sightline to the display itself was blocked and thus the participants could *only* view the display via the mirrors). Given the viewing distance, the final resolution was 1566.7 pixels/deg (thus allowing the measurement of vernier- and stereo-thresholds in the range of tens of arc seconds). The mean luminance of the visual display, measured through the mirrors and shutter glasses, was 3.85 cd/m^2^.

### Stimuli

**Fig 2:**
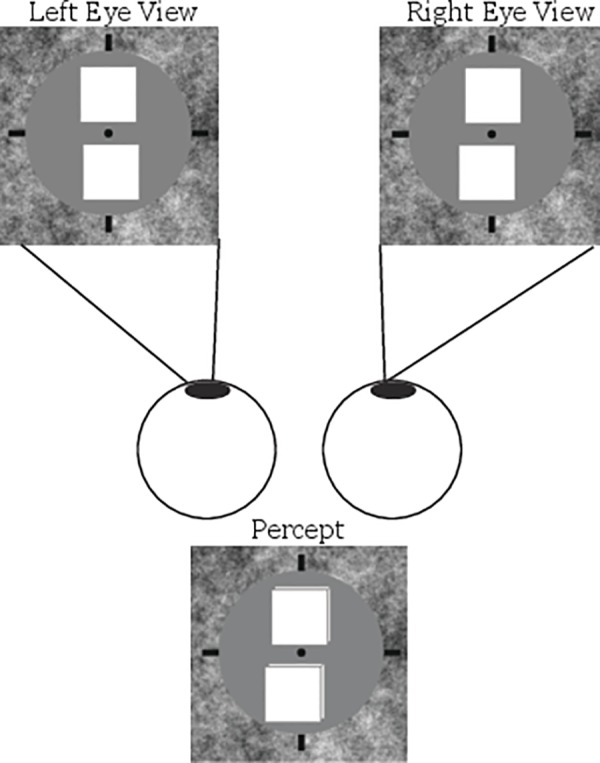
Example display (vertically oriented stimuli). On each trial participants were presented with two white squares offset from one another in both the z- and x-dimensions. Participants performing the vernier acuity task were asked to judge which of the two squares was further to the left, while participants performing the stereo acuity task were asked to judge which of the two squares was closer in depth. *Again*, *note that the actual stimuli were flat squares–they are presented as 3D boxes here for demonstration purposes*.

On each trial observers were presented with two 12 arcmin wide white squares for 1 s. Depending on the condition (see below), the squares could be positioned in one of two orientations—vertical or horizontal. In conditions utilizing a vertical orientation (see Fig 2), the two squares were centered around fixation and were separated from one another by 5 minutes of arc along the vertical axis. In this condition, the squares were offset from one another in both the horizontal and the depth dimensions. The offset distance was drawn independently for each dimension on each trial from a uniform distribution of 0 to 60 seconds of arc. The setup for conditions utilizing a horizontal orientation was identical, with the exception that the squares were centered around fixation along the horizontal dimension and were offset in both the vertical and depth dimensions.

Note that the two tasks, vernier acuity and stereo acuity, both depend on the judgment of small spatial offsets. The only difference is that in the case of vernier acuity the observer can judge the spatial offset of the two corresponding squares within the same eye, whereas in the case of stereoacuity, the observer needs to judge the spatial offset of corresponding squares between the two eyes.

Vergence was facilitated by a 1/f noise background pattern. In addition, a small fixation dot (27.5 arcsec diameter) was presented in the center of the display and nonius lines were presented near the edges of the background pattern to help the observer maintain fixation and monitor vergence during the experiments.

### Procedure

The entire experiment consisted of 1) a pre-test session where performance on all 4 combinations of task (vernier/stereo) and orientation (horizontal/vertical) were assessed, 2) fifteen blocks of training, and 3) a post-test session (identical to the pre-test session). Pre- and post-test sessions were conducted on separate days from any training blocks. During training, 3 training blocks were typically run per day (approximately 1 hour) so that each participant completed the experiment over a span of no more than 10 total days.

### Pre- and post-test assessments

In the pre- and post-test assessments, participants underwent four blocks of 500 trials each, fully-crossing the two acuity (vernier and stereo) and orientation (horizontal and vertical) conditions.

Vernier Acuity: In one block the squares were in the horizontal orientation, while in the other block the squares were in the vertical orientation. When the squares were in the horizontal orientation, the observers were asked to indicate which of the two squares was shifted more toward the top of the display. When the squares were in the vertical orientation, the observers were asked to indicate which of the two squares was shifted more toward the left of the display.

Stereo Acuity: Again, in one block the squares were in the horizontal orientation, while in the other the squares were in the vertical orientation. In both blocks, observers indicated which of the two squares appeared closer in depth.

The order in which observers completed the four blocks was pseudorandom between subjects; however, individual subjects completed the pre- and post-tests in the same order. Importantly, in an attempt to reduce the amount of learning from the tests themselves, no feedback was given during these assessments.

### Training

For the training phase, participants were each assigned one of the four orientation/task combinations (vertical/vernier: 4 participants, horizontal/vernier: 3 participants, vertical/stereo: 6 participants, horizontal/stereo: 3 participants). Each participant completed five sessions of training on their assigned condition, with each session consisting of three blocks of 500 trials (7500 training trials in total). During the training phase, auditory feedback was given following the observer’s response on each trial.

### Assessment

All of our major hypotheses rested upon differences in behavior in the pre- and post-test assessments. Thus, f0r each of the 8 pertinent blocks of data (i.e. 2 tasks–vernier/stereo; 2 orientations–vertical/horizontal; 2 tests–pre-test/post-test) we fit the proportion of clockwise responses *(P(CW))* to a dynamic logistic function of offset (*x*) with the slope parameter β1 evolving linearly over time (*t*) (and a constant bias term β0). In our previous work we have seen that this dynamic logistic function provides a significantly better fit to psychophysical data than standard approaches (i.e. fitting separate logistic functions to many individual blocks of data) when taking into consideration the number of free parameters [24]. We report the median 75% threshold given via bootstrap fitting of each participant’s data (see Eq 1).

$$P(CW)=\frac{1}{1+e^{\left(\beta_{0}-\beta_{1}\cdot x\right)}}$$ (1)

with *β*_1_ = *χ* + *α* ⋅ *t*

We note that most participants showed little change in performance within blocks (as was our goal–i.e. by not using feedback during pre-test/post-test blocks). Thus, in these participants the dynamic fit we obtained was roughly equivalent to a standard static logistic fit. However, we found that the dynamic fitting procedure was far superior for a few participants who appeared to have initial task confusion in some blocks (i.e. if participants had initial button confusion, a static logistic function would model their behavior as overall incredibly poor–essentially unfittable—while the dynamic procedure would model their behavior as initially poor/reversed, but eventually within the normal range). Thus, while the qualitative pattern of results was identical in the two approaches, the static approach necessitated dropping those participants who had one poor fit (with the reduction in power causing some analyses to fall just above p = .05), while the dynamic procedure allowed us to utilize the full set of participants and thus have fully powered analyses (see S1 Fig).

After each block was fit, for each combination of task and orientation, we computed a learning score (*LS*)

$$LS=\frac{\theta_{pre}-\theta_{post}}{\theta_{pre}}\;\times \;100\%$$

which reflects the difference between the pre-test threshold (more specifically the 75% correct threshold estimate given by the dynamic logistic function on the final trial of the given pre-test block, which we denote: θ_pre_) and the post-test threshold (again, the 75% correct threshold estimate given by the dynamic logistic function on the final trial of the given post-test block, which we denote: θ_post_) normalized by the pre-test threshold (and multiplying by 100 to reflect a percentage rather than a proportion).

**Fig 3:**
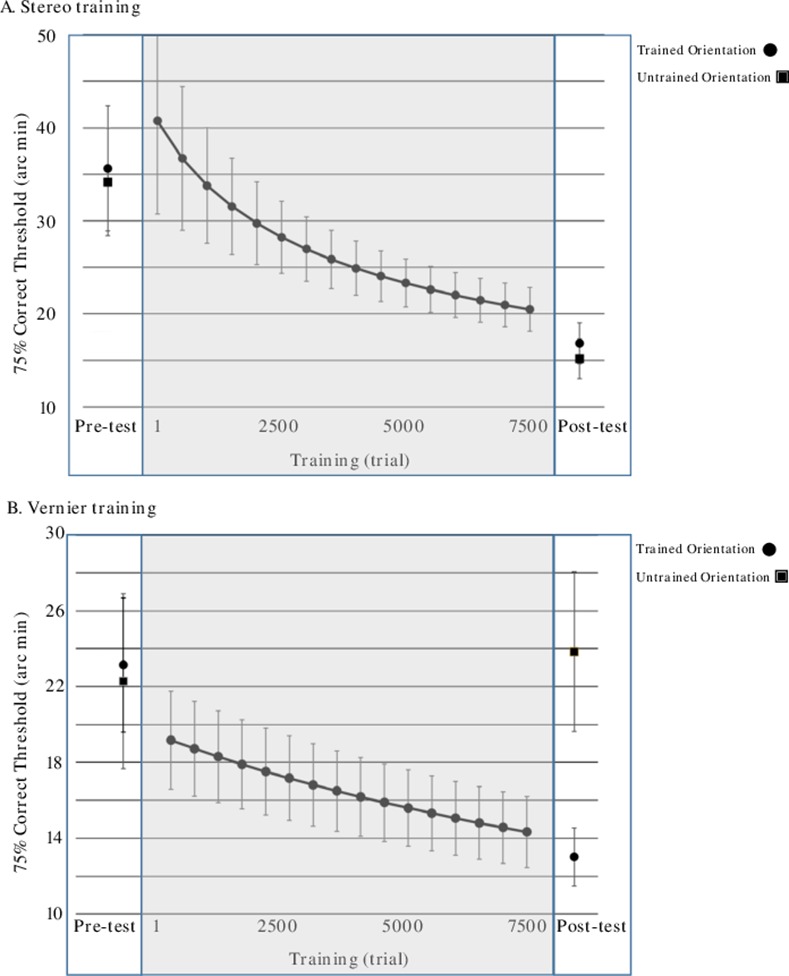
Raw performance on the trained task. **A) Stereo training:** Participants showed roughly equivalent performance on the two possible stimulus orientations before training commenced (“pre-test”). They then showed clear learning throughout the 7500 trials of training (“training”–gray region). And at post-test, they showed roughly equivalent improvements on the two possible stimulus orientations after training (“post test”). Consistent with our hypothesis, this suggests significant orientation transfer in the stereo trained group–a pattern that is quantified below (see Results/Fig 4). **B) Vernier training:** Participants showed equivalent performance on the two possible stimulus orientations before training commenced (“pre-test”). They then showed clear learning throughout the 7500 trials of training (“training”–gray region). However, during post-test, while they showed clear improvement on the trained orientation, there was little evidence of learning on the untrained orientation (“post test”). This suggests significant orientation specificity–a pattern that is quantified below (see Results/Fig 4). *Error bars denote s.e.m.

We utilized a ratio-based learning score because initial stereo performance was worse than initial vernier performance on average. Therefore, there would be more opportunity to learn in the stereo condition and thus more opportunity to transfer learning in the stereo condition based on a pure subtraction measure. This in turn could have significant and unfairly biased the results in the direction of our predictions (see Fig 3 for raw threshold values). The LS calculation is also more natural to interpret than a strict ratio (i.e. “0” in this value means no learning, positive values reflect percent improvement relative to baseline/pre-test).

## Results

### Improvements on the Trained Task & Orientation

**Fig 4:**
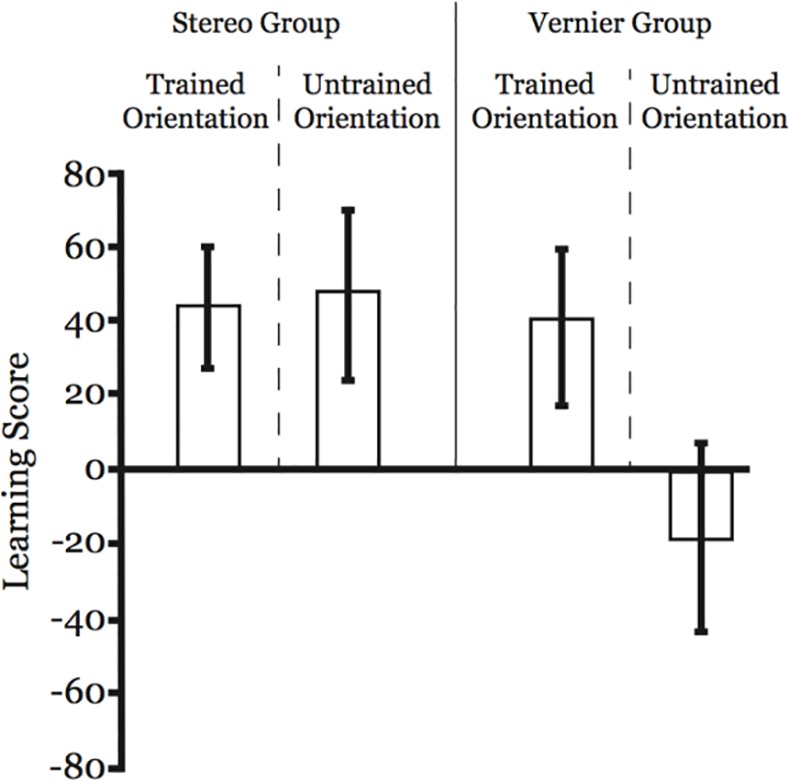
Learning and orientation transfer data. Both the stereo and vernier trained groups significantly improved their performance on their respective trained task/orientation combinations (left panel for each). However, while the vernier trained group showed evidence of clear orientation specificity (i.e. no improvement on the untrained orientation–far right panel), this was not the case for the stereo group, where the amount of improvement in the untrained orientation was similar to that seen for the trained orientation. *Error bars reflect 95% CI

As our question of interest pertains to differences in transfer of learning, it is first necessary to demonstrate that the participants showed clear learning on the tasks on which they were trained. To this end we ran a simple ANOVA using the learning scores for each participant’s individual trained task/orientation combination (i.e. the pre-test and post-test blocks that matched the trained task and orientation) with task (vernier/stereo) as a between-participants factor. In this particular analysis, the value of interest is the model intercept (i.e. given the way our learning score was calculated, a score of zero would indicate no learning, while positive values would indicate improved performance). The intercept value was statistically significant–*F*(1,14) = 48.6, *p* < .001, η_p_² = 0.78—indicating that participants did indeed improve at their trained task/orientation. Furthermore, the main effect of trained task was not significant–*F*(1,14) = 0.12, *p* = .74, η_p_² = 0.008 –indicating that the tasks were well equalized in terms of total percentage improvement via learning (Stereo: 43.54 +/- 9.05; Vernier = 39.45 +/- 6.75; see Fig 4). We also followed up this ANOVA with independent one-sample t-tests for each training task comparing the observed improvements against zero. Both of these tests resulted in statistically significant effects being observed, again indicating clear learning in both groups (Stereo: t(8) = 4.8, p = .001; Vernier: t(6) = 5.8, p = .001).

### Transfer of learning—Within task, across orientation

Our hypothesis was that there would be significantly greater orientation specificity seen in the vernier trained participants than in the stereo trained participants. To examine this, we conducted a 2 (orientation: trained/untrained) x 2 (task: vernier/stereo) ANOVA on the relevant learning scores. In this analysis we found main effects of both orientation, *F*(1,14) = 6.3, *p* = .025, η_p_² = 0.31, wherein learning was overall greater for trained orientations than untrained orientations, and task, *F*(1,14) = 14.1, *p* = .002, η_p_² = 0.50, wherein the stereo group showed overall more learning than the vernier group. However, and more critically for our hypothesis, a significant interaction between orientation and task *F*(1,14) = 8.3, *p* = .012, η_p_² = 0.37, was observed, driven by the fact that the stereo group showed roughly equivalent learning scores for both the trained and untrained orientations, while the vernier group showed learning only on the trained orientation (see Fig 4). This was confirmed via post-hoc t-tests separated by task. While no significant difference between the trained and untrained orientations was observed for the stereo trained group (trained: 43.5 +/- 9.1; untrained: 47.5 +/- 10.1; t(8) = 0.25, p = .81), a significant difference was observed for the vernier trained group (trained: 39.5 +/- 6.7; untrained: -17.2 +/- 12.3; t(6) = 4.61, p = .004). In other words, as has been consistently reported in the visual training literature, observers trained on the vernier acuity task showed training gains that were specific to the trained orientation. Meanwhile, consistent with our read-out based hypothesis, observers trained on the stereo acuity task showed training gains that transferred to the orthogonal orientation.

### Transfer of learning–Across tasks

Our a priori expectations were that we would see limited to no inter-task transfer. Indeed, the simplest discriminant that could be used to solve the vernier task (whether it was horizontal or vertical) should be of little benefit to the stereo task. Likewise, the discriminant solution to the stereo task should be of little benefit to either vernier task. We conducted a 2 (orientation: trained/untrained) x 2 (task: stereo/vernier) ANOVA on the untrained task performance. A non-significant intercept in this model indicated that overall there was not a significant difference from zero (i.e. no evidence of overall learning; *F*(1,14) = 0.11, *p* = .74, η_p_² = 0.008). And while numerically the vernier trained group showed more improvement on the untrained task than the stereo trained group (vernier = 12.8% +/- 16.2; stereo = -5.6% +/- 14.3) neither the main effect of task nor the main effect of orientation approached significance (both *F*’s < 1, both *p*’s > .4). Thus, overall we saw no evidence of cross-task improvements.

## Discussion

As has been repeatedly observed throughout the perceptual learning literature, observers trained on a vernier discrimination task in one orientation showed no evidence of transfer of learning to the orthogonal orientation. However, observers trained on a stereo discrimination task using the exact same stimuli showed evidence of significant transfer to the orthogonal orientation. These results are not consistent with beliefs that perceptual learning is necessarily specific to orientation. Instead, this pattern of results is consistent with the emerging view that perceptual learning is significantly mediated by higher-level integration areas that learning rules for dividing the perceptual space. These rules, depending on the exact transformations that are learned, may then be transferable to new cases. In the case of the vernier task, the simplest rule that could be learned corresponds to a discriminant in the image plane (i.e. that divides the space into up/down or left/right). When the stimuli are rotated by 90°, this rule is no longer applicable and thus the learning appears specific (i.e. if one has learned to divide the world into up/down, this is of little use for a task that requires the world to be divided into left/right). Conversely, in the stereoacuity task, the simplest rule that could be learned corresponds to a discriminant in the depth plane (i.e. closer/farther). This rule is independent of the stimulus orientation in the image plane and thus nearly full transfer would be expected (and was found). It is important to note that our hypothesis is not that “stereo training transfers,” while “vernier training does not transfer.” Instead, our belief is that the representation learned during stereo training were sufficient to allow for transfer across changes in orientation in the image plane. We have no belief though that this representation would be sufficient to, for instance, transfer across positions. Similarly, we might expect vernier training to transfer to one other orientation (a rotation of 180°—in other words, asking participants to change their response mapping in order to respond to the other direction) as the learned representation should be sufficient to perform this new task.

Previous work has shown that monocular and dichoptic vernier acuity, where the elements of the vernier stimulus are either shown to the same or different eyes, share a common mechanism, and has argued that the locus of hyperacuity vernier judgments must thus lie at the cortical level where input from the two eyes is combined [25]. These findings make it unlikely that improvements in vernier- and stereo-acuity depend on separate perceptual learning mechanisms, where vernier acuity depends on neural changes early in visual cortex prior to binocular combination, and improvements in stereo-acuity depend on changes later in the visual stream.

Our design used relatively low luminance stimuli due to the use of shutter goggles that attenuated the screen’s brightness. The benefit of shutter goggles relative to more traditional stereoscopes is that the potential for binocular misalignment is greatly reduced, which especially benefits experiments with naïve observers. Previous work has found that both vernier- and stereo-acuity are relatively insensitive to luminance for the type of full contrast, long duration stimuli we employed here [26], and we therefore expect our results would hold for other luminance levels.

We note that these results are somewhat different from previous work in perceptual learning [6] where both stereo and vernier acuity training was found to be orientation specific. While the current experiment differs in a number of ways from this past work (e.g. length of training, exact stimuli, etc), perhaps most critical is the overarching design type. Specifically, our design, unlike the previous work, utilized a pre-test→training→post-test design. Thus, our participants had some limited amount of exposure during pre-test to the transfer orientations–exposure that may be critical to producing transfer [13]. However, we would note that this exposure was not sufficient to produce orientation transfer in the vernier acuity trained group, and thus it is not a wholly sufficient explanation for the transfer that is observed in the stereo acuity trained group. Our task also utilized offsets in both dimensions for all participants. Thus, stereo participants viewed offsets in the respective vernier dimensions during training and the vernier participants viewed offsets in the stereo dimension during training. This variation was the same across all participants, however, it nevertheless remains possible that this aspect of the experimental design played a role in the dissociation seen in the transfer effects across groups (i.e. depends on the difference in the sensitivity to spatial overlap between the tasks). This is potentially important as dependence on spatial overlap could potentially be indicative of changes in lower-level areas. Pinning these relationships down further will require future work to utilize both our setup as well as stimuli that do not differ along both dimensions (i.e. participants will not observe exactly the same stimuli; additional measures to eliminate confounds related to spatial overlap).

While our results obviously do not rule out a low-level contribution to perceptual learning effects (and indeed, there is evidence for such effects—[27]), they do add to a growing body of behavioral research suggesting a clear role for higher-level areas in perceptual learning–even in those paradigms that produce stimulus-specific learning.

## Supporting Information

S1 Fig Modeling participant performance. A) For the vast majority of participants, estimates of threshold levels in the pre- and post-test blocks were reasonably equivalent in the dynamic logistic and the static logistic data fitting approaches. This is not surprising given that no feedback was provided during these blocks and thus little learning would be expected. The left panel shows the dynamic approach (logistic moving from early in the task represented by blue colors to late in the block represented by yellow colors) and the static approach (represented by black line). Essentially no change is seen in the logistic function in the dynamic approach and it overlaps completely with the static approach. This can also be seen in the 75% thresholds plotted in the right panel (where the blue line represents the dynamic approach and the black the static approach). B) For a small number of participant/block combinations however, the dynamic approach offered a much better fit to the data. In these cases participants typically started incredibly poorly (perhaps due to uncertainty regarding instructions, etc), but quickly demonstrated thresholds in the expected range (i.e. given the incredibly poor initial performance and incredibly fast change toward improved performance this is unlikely to be “learning”–but is more likely related to changing response mappings). This can be seen in the left panel where the dynamic logistic starts (blue colors) essentially flat, but quickly evolves (moving toward yellow colors) to a more reasonable psychometric function. Similarly, in terms of threshold, the static approach drastically misestimates the actual capability of the participant.
(TIF)

S1 Data Supplemental Data. **Individual performance.** Estimated 75% thresholds for vernier and stereo discriminations in the pre- and post-tests and on the first and last trial of training on the respective task (Training Group).
(XLSX)
