# Supplementary figures and images for: Orientation Transfer in Vernier and Stereoacuity Training
Source: PLoS One. 2015 Dec 23;10(12):e0145770. doi: 10.1371/journal.pone.0145770 (PMC4689363; doi:10.1371/journal.pone.0145770)

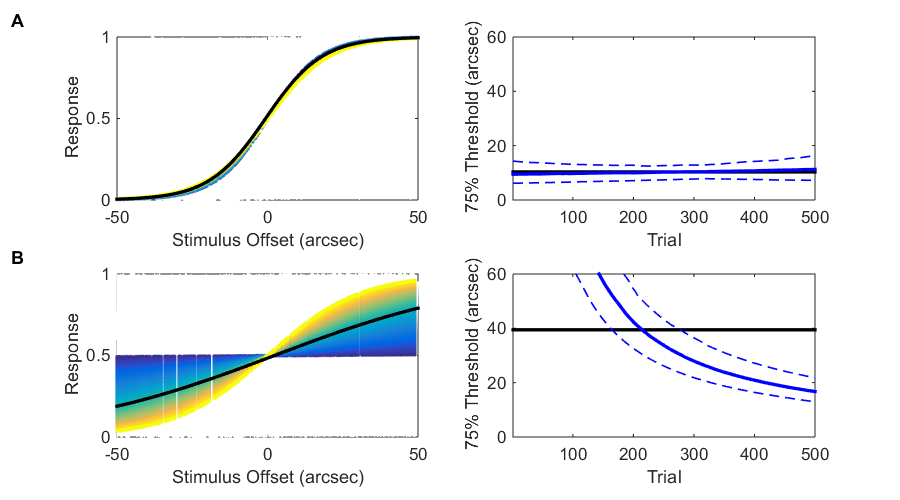

Supplement: S1 Fig — A) For the vast majority of participants, estimates of threshold levels in the pre- and post-test blocks were reasonably equivalent in the dynamic logistic and the static logistic data fitting approaches. This is not surprising given that no feedback was provided during these blocks and thus little learning would be expected. The left panel shows the dynamic approach (logistic moving from early in the task represented by blue colors to late in the block represented by yellow colors) and the static approach (represented by black line). Essentially no change is seen in the logistic function in the dynamic approach and it overlaps completely with the static approach. This can also be seen in the 75% thresholds plotted in the right panel (where the blue line represents the dynamic approach and the black the static approach). B) For a small number of participant/block combinations however, the dynamic approach offered a much better fit to the data. In these cases participants typically started incredibly poorly (perhaps due to uncertainty regarding instructions, etc), but quickly demonstrated thresholds in the expected range (i.e. given the incredibly poor initial performance and incredibly fast change toward improved performance this is unlikely to be “learning”–but is more likely related to changing response mappings). This can be seen in the left panel where the dynamic logistic starts (blue colors) essentially flat, but quickly evolves (moving toward yellow colors) to a more reasonable psychometric function. Similarly, in terms of threshold, the static approach drastically misestimates the actual capability of the participant. (TIF) [file pone.0145770.s001.tif]
